# Sexual communication patterns of heterosexual-identified men who have sex with men

**DOI:** 10.1177/02654075251377141

**Published:** 2025-09-04

**Authors:** Travis R. Scheadler, Megan W. Rowe, Salem Rao, Oliver W. J. Beer, Tyrone J. Curtis, Sandra Kwan, Shih-Ju Claire Lung, Adam Busch, Daniel Vandervoort, Paul A. Shuper, Andrew D. Eaton

**Affiliations:** 1 School of Social Work, University of North Carolina at Charlotte, USA; 2 6846 Faculty of Social Work, University of Regina, Canada; 3 Faculty of Health, Dalhousie University, Canada; 4 6633 School of Health Professions, University of Plymouth, UK; 5 8205 School of Public Health and Social Policy, University of Victoria, Canada; 6 7938 Department of Psychiatry, University of Toronto, Canada; 7 HQ Toronto, Canada; 8 7978 Centre for Addiction and Mental Health, Canada; 9 Jane Addams College of Social Work, University of Illinois Chicago, USA

**Keywords:** Heterosexual-identified men who have sex with men, sexual communication, sexual interests, sexual script theory, consent

## Abstract

The sexual communication patterns that comprise how heterosexual men with concordance between identity and behavior navigate their sexual encounters with women are well-established. Some heterosexual men experience discordance between their sexual identities and behaviors and may have unique practices of sexual communication, but this has not been studied. Knowledge of their sexual communication practices is an important step toward understanding and improving their sexual experiences. Therefore, semi-structured interviews were conducted with 11 heterosexual-identified men who have sex with men to learn more about their sexual communication strategies. Three themes were identified through interpretative phenomenological analysis: meeting sex partners, clear communication with men, and poor communication with women. Findings suggest that sexual communication is gendered. Participants reported utilizing short and explicit verbal and written communication strategies with other men via the internet and in various sexualized venues. Yet, participants more frequently used implicit sexual cues as the primary source of sexual communication when with women. Implications related to boosting sexual satisfaction and preventing nonconsensual sexual encounters are discussed.

## Introduction

Most heterosexual men experience concordance between their sexual identity, attractions, and behaviors, marked by identifying as heterosexual, reporting attractions toward women, and only engaging in sex with women. However, an estimated .5–3.5% of heterosexual men have sex with men and 1.26–5.4% of men who have sex with men (MSM) identify as heterosexual (Scheadler et al., 2024). Heterosexual-identified men who have sex with men (H-MSM) have shared many reasons justifying or dismissing their sexual identity-behavior discordance. Some say sex with men is infrequent or accidental (Reback & Larkins, 2010) and some conceptualize it transactionally as an exchange for money or drugs (Fernández-Dávila et al., 2008). Importantly, scholars argue that challenging the heterosexual identity of H-MSM may discourage them from engaging in research and accessing healthcare (Scheadler et al., 2024). Accepting that heterosexuality may be flexible and not rigidly defined by conformity to sexual identity-behavior concordance is an important step toward building a stronger understanding of H-MSM.

Research on H-MSM is limited. A recent scoping review revealed H-MSM have hypermasculine personas and attitudes, conceal their same-sex sexual encounters from most of their networks, are more often the insertive partner during anal intercourse with other men, and lack emotions for or have negative emotional responses to men (Eaton et al., 2023, 2025a). However, research has not yet explored the sexual communication patterns of H-MSM. Gaining knowledge of the sexual communication patterns of H-MSM is an important next step toward understanding the sexual experiences of H-MSM. Sexual communication is important for many areas of sexual health and well-being, including sexual satisfaction (Mark & Jozkowski, 2013), sexual functioning (Mallory et al., 2019), engagement in risky sexual behaviors (Ayer et al., 2021), and adherence to HIV treatment (Jin et al., 2021). Thus, the present study aimed to examine the sexual communication patterns of one sample of H-MSM.

### Sexual communication among concordant heterosexual men

Sexual communication encompasses both verbal and nonverbal exchanges about sexual preferences, boundaries, and desires between partners. Research indicates that effective sexual communication is linked to higher levels of sexual satisfaction and relationship quality, as it facilitates mutual understanding and consent (MacNeil & Byers, 2005, 2009). Although H-MSM experience sexual identity-behavior discordance, they still adhere to societal dominant discourse on masculine norms like other heterosexual men (Eaton et al., 2025a). Both H-MSM and other heterosexual men often feel a need to prove and maintain a heterosexual and masculine image (Eaton et al., 2025b; Knight et al., 2012). Likewise, there are possibilities that sexual communication patterns of H-MSM are similar to those of other heterosexual men.

Importantly, gender power dynamics are one of the most influential factors in sexual communication patterns among heterosexual men and their sexual partners (Waling, 2024). For instance, societal narratives around men’s sexuality often emphasize that men possess inherently high sex drives and are expected to frequently desire and initiate sexual activity (Morrison et al., 2015), reinforcing the stereotype that they might be more readily willing for sexual encounters than women (Masters et al., 2013; Murray, 2018).

Also, heterosexual men often attend to nonverbal sexual cues to determine and/or confirm the sexual interests of women. For example, heterosexual men often rely on subtle, nonverbal cues based on eye contact, body language, and/or facial expressions from partners to assess sexual interest and when seeking consent, especially during casual sexual encounters (Waling, 2024; Willis et al., 2019). Heterosexual men are likely to interpret implicit sexual cues as a form of consent (Jozkowski et al., 2018; Newstrom et al., 2021). Heterosexual men’s reliance on their interpretations of implicit sexual cues opposed to explicit verbal communication can be problematic and may lead to nonconsensual sexual interactions (Jozkowski et al., 2018). There are possibilities, though, that sexual communication patterns of H-MSM may not align with sexual communication patterns of other heterosexual men. H-MSM might utilize verbal and explicit sexual communication strategies when engaging with potential partners. Because H-MSM differ from other heterosexual men in their sexual interactions with men, it is important to also develop an understanding of the sexual communication patterns of sexual minority men.

### Sexual communication among gay and bisexual MSM

Although H-MSM are not simply closeted gay or bisexual men (Eaton et al., 2025a), drawing on research about gay, bisexual, and other MSM can provide a valuable theoretical foundation for understanding same-sex sexual communication. Beres et al. (2004) argued sexual communication between non-heterosexual partners differs from that of heterosexual partners due to the distinct ways in which sex and gender dynamics influence sexual communication patterns. Findings demonstrated gay men entail greater flexibility around dominant initiating and gatekeeping roles that are generally ascribed to male and female heterosexual sex partners, respectively (Beres et al., 2004). However, sexual minority men, like heterosexual men, rely heavily on nonverbal sexual communication (Beres et al., 2004; Webber et al., 2024). Similar to heterosexual men, Marcantonio et al. (2022) found the use of explicit verbal versus nonverbal sexual communication varied depending on the type of sexual activity and relationship context. For oral sex, participants most frequently used explicit nonverbal signals, a lack of response, or explicit nonverbal consent (Marcantonio et al., 2022). In contrast, explicit verbal consent was more commonly reported for penetrative or anal sex (Marcantonio et al., 2022). Additionally, MSM were more likely to use explicit nonverbal, implicit verbal, and implicit nonverbal cues in casual relationships (Marcantonio et al., 2022).

These findings extend to virtual dating spaces. Epidemiological evidence suggests gay and bisexual men utilize technology for dating more than individuals of other genders and sexualities (Goldenberg, 2019). The immediacy provided by virtual platforms has facilitated more direct exchanges about sexual desires, preferences, and boundaries (Suenzo, 2024). Yet, just as sexual communication styles differ in physical spaces, they also vary across virtual platforms, influenced by each platform’s norms and communication styles. Specifically, Suenzo (2024) noted that certain platforms, such as Grindr, permit users to utilize fake names and faceless photos allowing discretion. Notably, while conversations on Grindr are often more direct, conversations on Instagram or WhatsApp might be more “playful and spontaneous – [offering a] way to keep romantic and sexual interactions engaging” (Suenzo, 2024, p. 11). As such, certain spaces where H-MSM might meet prospective sexual partners may facilitate more explicit verbal and written sexual communication. An understanding of sexual script theory may help to understand how and why H-MSM might adopt various sexual communication practices.

### Sexual script theory

The sexual communication patterns of H-MSM can be examined through sexual script theory, which posits human sexual behavior is socially constructed based on accepted norms for how individuals should act or behave (Simon, 2017). Simon and Gagnon (1984) identified three levels of sexual scripts informing sexual behavior: cultural, interpersonal, and intrapersonal. Sexual cultural scripts drawn from the law, education, religion, and other cultural forces determine which sexual behaviors are encouraged and which are illegal, stigmatized, and/or discouraged (Gagnon, 1990; Simon, 1996; Wiederman, 2015). Interpersonal sexual scripts are important for sexual behavior. Specifically, each social actor adapts what they know about sexual norms to each unique situation, requiring social actors to adjust and improvise to each situation (Simon & Gagnon, 1984). This can result in harmonious interactions when two or more social actors have similar scripts, including their beliefs, attitudes, and desires related to sex. However, this can result in conflict when two or more social actors have sexual scripts that do not converge (Wiederman, 2015). Meanwhile, intrapersonal sexual scripts involve memories, fantasies, and mental rehearsals wherein individuals think about their sexual behaviors and decide on how they will act (Simon & Gagnon, 1984).

Sexual scripts are gendered in extant research. Early studies guided by sexual script theory concluded the socialization of boys emphasizes hypermasculinity and aggressiveness toward girls and women (Byers, 1996; Mosher & Tomkins, 1988). Cisgender heterosexual men who embrace masculinity and cisgender women who express high levels of femininity are more likely to share attraction and choose one another as partners, highlighting the role of gender presentation (Tillewein et al., 2022). In their narrative review, Dangerfield et al. (2017) discussed that masculinity stereotypes, which influence perceived norms related to power, impact sexual positioning and condom use among MSM and men who have sex with men and women (MSMW). Being the insertive partner is perceived to be associated with hypermasculinity whereas being the receptive partner is perceived to be associated with femininity (Reback & Larkins, 2010). As such, being the insertive partner allows MSM to conform more closely to expected norms around what it means to be a man. Likewise, H-MSM tend to embrace hypermasculinity, partially by being the insertive partner during anal intercourse with other men and by concealing their same-sex sexual behaviors to avoid being perceived as gay or feminine and to be perceived as a masculine man (Eaton et al., 2025a). Thus, a combination of cultural, interpersonal, and intrapersonal sexual scripts influence how H-MSM engage in sex with other men.

Relatedly, sexual scripts indicate men, including H-MSM, are expected to be sexually dominant while women are expected to be submissive and obedient (Sanchez et al., 2012). Men hold sexual agency, marked by high sex drives and the ability to initiate sexual activity (Wiederman, 2005) while women are expected to be relationship-driven rather than sex-driven (Levant et al., 2012). Moreover, Schnarrs et al. (2012) reported MSMW are attracted to aggressiveness in other men and view a man who is the receptive partner during anal sex as participating in the role of a woman, suggesting MSMW perpetuate gendered sexual scripts with both men and women. Schnarrs et al. (2012) also noted MSMW feel more emotionally fulfilled by women but more sexually fulfilled by men and that men are encouraged to express themselves sexually while women are expected to be more reserved. Participants shared being more sexually adventurous with men was accepted while certain sexual acts, such as allowing a woman to insert a finger or toy into a man’s anus, was degrading for men, highlighting how sexual scripts play important roles in how MSMW engage in sex with men and women (Schnarrs et al., 2012).

Further, research suggests heterosexual men disapprove of sexual assertiveness in women (Klein et al., 2019). Yet, heterosexual men have been critical of gendered sexual scripts (Murray, 2018). They also have reported feigning sexual desires to comply with traditional gendered sexual scripts to demonstrate high degrees of masculinity (Murray, 2018). Thus, traditional gendered sexual scripts may make men uncomfortable to have open conversations about sex with women while facilitating comfort for men to have open conversations about sex with men. Sexual communication, though, is important for improving sexual satisfaction (Mark & Jozkowski, 2013) and preventing unwanted sex, especially as adherence to traditional sexual scripts is related to engaging in coercive or unwanted sex (Rittenhour & Sauder, 2024). Newstrom et al. (2021) added women view direct forms of communication as necessary for consent whereas men believe indirect communication strategies can be interpreted as someone providing consent. That is, traditional sex scripts discourage communication about sexual desires and consent, permitting men to initiate sex with limited or no explicit verbal communication.

### The current study

Importantly, identifying as heterosexual but engaging in same-sex sexual acts defies common sexual scripts. Although research has begun to examine the sexual identities, behaviors, and attractions of H-MSM (Eaton et al., 2025a), less is known about the communication patterns H-MSM use to discuss sex and sexuality with their sexual partners. Perhaps, H-MSM may be more sexually adventurous and communicative with other men than they are with women. Indeed, heterosexual men often adhere to traditional gendered sex scripts even when they are critical of them (Murray, 2018). These traditional gendered sex scripts may pressure H-MSM to limit communication about sex when with women but may permit H-MSM to freely discuss sexual desires and consent when with men, especially if they meet and interact with these men in sexualized environments that seem to facilitate explicit verbal and written sexual communication. However, research examining the sexual patterns of H-MSM remains scarce. In fact, no known studies have explored the sexual communication patterns of H-MSM, though such knowledge may have implications related to sexual satisfaction and coercive sex. An investigation into the sexual communication strategies of H-MSM will provide greater insights into understanding and supporting this population. Therefore, the purpose of the present study was to explore the sexual communication strategies of H-MSM.

## Method

The present study utilized interpretive phenomenological analysis (IPA), a recommended methodology when working with understudied and marginalized populations (Creswell & Poth, 2018). IPA centers the participants in knowledge generation, emphasizing detailed personal lived experiences within specific sociocultural contexts and highlighting how experiences across individuals can both converge and diverge (Smith et al., 2009). In addition, instead of building theory, IPA focuses on interpreting the experiences of individuals (Smith & Fieldsend, 2021). IPA aims to understand how individual participants make meaning of particular experiences. Further, both IPA and sexual script theory recognize the influence of cultural, interpersonal, and intrapersonal dynamics (Simon & Gagnon, 1984; Smith et al., 2009). Thus, IPA was a suitable approach for exploring the sexual communication patterns of H-MSM, an understudied population. Institutional ethics approval was obtained from the University of Regina’s Research Ethics Board (Protocol #2022-083) and the University of Toronto’s Research Ethics Board (Protocol #43473).

### Paradigm and positionality

In alignment with IPA, the research team adopted an interpretivist-constructivist paradigm. Researchers using an interpretivist-constructivist paradigm recognize there is no universal truth but many possible realities (Guba & Lincoln, 1994). That is, individuals can each live in their own truth based on their own experiences, attitudes, and beliefs. Through this paradigm, researchers believe knowledge is co-constructed by participants and researchers and the job of the researchers is to make sense of what participants share (Smith et al., 2009). Interpretivist-constructivist researchers are tasked with analyzing and contextualizing the experiences of participants based on prior knowledge and sociocultural factors (Smith et al., 2009). To do so, researchers must focus on affording participants a safe and affirming environment where they are comfortable voicing their lived experiences (Alase, 2017), which is particularly important when working with H-MSM who often conceal their identities and experiences (Scheadler et al., 2024).

An important element in IPA and an interpretivist-constructivist paradigm is to recognize and bracket biases to minimize the researchers’ influence on sharing the lived experiences of participants (Alase, 2017). As such, our positionality emphasizes affirming and supporting H-MSM opposed to challenging their identity-behavior discordance. Most members of the research team have concordant sexual identities, behaviors, and attractions and a couple student members lacked prior experience or knowledge of H-MSM before beginning this project. Our team held regular meetings to discuss and bracket our biases and encourage acceptance and openness. Most members of the research team came from social work, psychology, and public health, which each emphasize affirming identities and experiences of others to achieve greater health equity (Jenkins & Johnston, 2004; Serchen et al., 2024). These mutual values allowed the research team to approach this study with openness. Moreover, although a couple student researchers had limited prior experience with H-MSM, community partners who identified with the community and/or had experience with the community offered their insight throughout each stage of the study to further minimize the risk of misinterpretations of the data.

### Participants and procedures

Participants included 11 cisgender men aged 18 and older who resided in Canada, identified as heterosexual, and reported engaging in sex with at least one other man. Eligibility was not limited to residents of any particular province in Canada; however, the city and province participants resided in were not recorded. Table 1 provides key demographic information about each participant along with their pseudonyms. Only two participants shared their religious belief system. Reticence to share demographic information is not surprising given the heightened worries related to privacy and anonymity among H-MSM (Scheadler et al., 2024). Small samples of relatively homogenous participants are desirable in IPA to maintain an idiographic approach (Alase, 2017; Smith et al., 2009). In fact, some scholars argue larger sample sizes are not necessary for IPA (Smith et al., 2009).

**Table 1.** Participant demographic information.

Pseudonym	Description
Alex	40–49, Chinese, christian, married
Ben	40–49, Black, catholic, single
Rajesh	30–39, Indian, married
Caleb	40–49, White, recently separated
Daniel	30–39, Black, single
Elijah	30–39, White, single
Gabe	30–39, White, in a relationship
Henry	30–39, White, single
Kevin	70–79, White, divorced
Noah	20–29, White, recently single
Quinn	40–49, Black, in a relationship

Respondents were recruited via paid advertisements on social media (e.g., Grindr, Snapchat), posts to online forums, and flyers in HIV clinics. These efforts were employed from December 2022 through November 2023. The recruitment material contained a link to a study website with details about the study including consent form, the researchers’ contact information and interviewers’ biographies, and a link to an interest form. Within 48 hours after completing the interest form, one of the interviewers (first and last authors) contacted prospective participants via email to schedule an interview. Interviews were scheduled at the participants’ earliest convenience, which was between 1 and 16 days after being contacted to schedule the interview. All interviews occurred via Zoom in English and lasted an average of 65.26 minutes (*SD* = 19.86). The consent form was reviewed, and oral consent was obtained prior to conducting the interviews. Data were collected between January 2023 and January 2024.

A semi-structured interview guide was used during the interviews. Questions and probes were designed to explore details about the sexual communication practices of the participants. For example, participants were asked “How do your sexual encounters with women differ from your sexual encounters with men?” and “How do you communicate about sex and sexuality in your relationships?” Following these questions, participants were probed to share details about navigating consent and identifying sexual stimuli and arousal with their partners. All interviews were audio-recorded with an initial transcript generated via natural language processing using NVIVO Transcription, and then the transcript was corrected by research assistants. Each participant received a $30 e-gift card to thank them for their participation.

### Data analysis

Guidelines for IPA coding set forth by Smith et al. (2009) were followed. Nine coders were provided training on how to conduct IPA coding. Then, each coder familiarized themselves with the interviews by reading and re-reading the transcripts and listening and re-listening to the audio files. After each coder practiced coding one transcript, the coding team met to discuss initial impressions as well as emergent codes and themes, and to critically examine our own biases and reactions. Doing so allowed each member of the research team to adopt a more inclusive view of masculinity that is nonjudgmental of sexual identity-behavior discordance and of the participants’ experiences. This meeting also was useful in helping the research team establish consistent verbiage while coding and obtain feedback from each other in how to effectively code the interviews.

Following this meeting, each coder was assigned at least one interview to code. Coders were instructed to code both the transcript and audio files via multimodal coding, which was useful for documenting changes in inflection or affect to develop a more comprehensive understanding of the participants’ experiences (Craig et al., 2021). In addition, each interview was independently coded by two coders to avoid groupthink. One member of the research team then collated all the codes together. Each coder was then instructed to independently review the codes and cluster the codes into themes and categories. The research team then met to review reflect on each coder’s theme structure. During this meeting, the research team identified similarities and differences across the various theme structures and then agreed upon a theme structure to be reported. The first author returned to the transcripts to ensure the themes accurately represented the participants’ experiences.

### Trustworthiness

Several strategies were employed to strengthen the trustworthiness of the study. First, members of the H-MSM community as well as expert clinicians with experience working with H-MSM were involved throughout the research process. These individuals assisted with conceptualizing and designing the study and analyzing the data. In addition, the use of multiple coders helped to prevent groupthink and increase the comprehensiveness of the codes and themes. Using multimodal coding also allowed the research team to more thoroughly understand the participants’ experiences (Craig et al., 2021). Also, as suggested by Smith et al. (2009), peer supervision was an important element in strengthening the trustworthiness of the study. According to Smith et al. (2009), supervision is important to ensure interpretations are based on the experiences shared by the participants and not by the researchers’ personal biases. Finally, the two interviewers wrote memos after each interview to record initial thoughts and document any biases, which was helpful in guiding team discussions and subsequent analyses.

## Findings

Three themes related to sexual communication were identified from the data analysis: meeting sex partners, clear communication with men, and poor communication with women. More details about each of these themes, along with exemplar quotes, are shared below.

### Theme one: Meeting sex partners

A key aspect of sexual communication is identifying potential sex partners. As such, the first theme in the present study centered around descriptions of how and where participants met and/or attempted to meet possible sex partners. At the intrapersonal level, participants reflected on their own availability and on locations that they know can provide easier access to potential sex partners. For example, participants met sex partners at cruising parks, bathhouses, saunas, hotels, short-term housing rentals, school, work, and in cars. Kevin, for example, shared, “When I’m busy and out of town, I cruise. I look for hook up places.” Cultural factors were also important for locating potential sex partners. That is, participants adapted to changing circumstances by seeking and communicating with potential sex partners through online dating and hook-up apps. Noah shared:

In my situation, I mean, I was online quite a bit, and I had recently ended a relationship in the early start of COVID. So that really influenced my sex patterns because I could find that sex would be a lot more, like, easier available online and especially like with men.

Interpersonal factors and intrapersonal values were also important when using these social media platforms to communicate plans for where to physically meet prospective sex partners. They often met at either their house or their sex partner’s house. Daniel stated, “I can go to your apartment, [or] you can come over to mine,” signifying that they were flexible on the location and could adapt to what the other individual desired. Intrapersonally, many participants valued discretion and aimed to keep their personal and sex lives with other men separated. Therefore, most participants avoided inviting sex partners to their homes, choosing instead to travel to their sex partner’s home. For example, when asked why they prefer to travel to their sex partner’s house, Ben disclosed:

I live with my family members, so I think that’s one reason. And the other reason is, more importantly, I know who I am. I’m not going to disrespect someone’s house ever, right? I’m of that mindset. It’s like, okay, someone else enters your house, you show them respect, you be respectful of their things, and whatnot have you, right? So, I know I’m going to conduct myself in someone else’s house, right? I will take off my shoes, like what I believe is good conduct in someone’s house. If I were to ever host, not saying I would, I can’t guarantee someone else’s behavior.

As illustrated in this example, Ben, like the other participants, implied he had multiple reasons for preferring to travel to his sex partner’s house. Specifically, participants valued politeness, safety of their personal possessions, and discretion (i.e., personal safety from the potential consequences of their families learning about their same-sex sexual behaviors).

### Theme two: Clear communication with men

The second theme revolved around the presence of clear and explicit communication about sex with other men albeit with the intrapersonal priorities discretion and privacy mentioned above. As such, participants reported conveying little information about themselves in their online profiles (e.g., on Grindr), waiting until interpersonal conversations began to communicate more about their sexual interests. This limited form of initial communication allowed participants to adapt to different interpersonal interactions and be selective about what they share with others and with whom they share it. Alex said, “the pictures [on my profile] are just the body picture, not the face picture” and Caleb said, “I don’t even really like when people ask for a picture exchange.” Noah also shared, “[my profile] is kind of blank.” Participants also used pseudonyms to protect their identities on dating and hook up apps. Elijah stated that they use a popular fictional character as their username and shared, “it’s funny how often people ask me if that’s my real name.” Notably, some participants listed some details about their physical characteristics, sexual interests, and intentions on their profile, encouraging interpersonal engagement by allowing other users to screen them for sexual compatibility. Rajesh, for example, shared:

I just share a little bit of what my intentions are, what I’m looking for, and my age, height, weight, and I think [the app] also asks for hairy, body type, something like that. And then my HIV status—it’s negative—so I just share that…I don’t show my pictures on my profile.

Although Rajesh shared more information on their profile than other participants, he remained discreet and shared only minimal details about himself.

After initiating conversations with other users, participants valued direct and short sexual communication via online platforms. Utilizing clear and concise sexual communication strategies, especially via online platforms, permitted participants to more rapidly identify possible sexual partners and determine their sexual compatibility. Quinn, for example, stated, “I usually ask what they want first and then communicate what I’m looking for and make sure that it works out.” Indeed, participants were straightforward in their conversations about sex with other men. Kevin shared examples of questions he asks other men to encourage clear sexual communication:

I’ll even ask, “what would you like to do? Do you like to tell me what you like, or would you like me to experiment?” And in a lot of cases—most cases—they’ll tell me [what they like], but there’s been the odd time that they’d say, “well, you know, go ahead and experiment and I’ll tell you if I like it or not.”

The normalization of explicitly discussing sexual desires coincided with explicitly discussing consent with male sex partners. More specifically, participants mentioned they ensured that fulfilling their own sexual desires (an intrapersonal sexual script) with the other person (an interpersonal sexual script) would be consensual. Gabe engages in more interpersonal communication, directly asking others about consent. They said, “That’s how I do it…‘Do you want to have a conversation about consent?’ Yeah, I mean, we talk about it. It’s a very frank discussion. And then, whatever happens after that happens.” Henry also explained:

I would say that of the conversations I have on Grindr, it’s like one percent of the conversations will actually lead to something happening. Either I’m not attracted, or I get a weird vibe, or they see my pictures and they take care of things themselves and then nothing ever comes of it. But in the instances where I do meet up with them, I make my intentions very clear and make it clear what it is that I’m looking for. And as long as they’re okay with that, like I kind of earmark that as consent. If I’m like, “hey, like, all I’m looking to do is meet up. I’m going to go down on you and then we’re going to part ways.” If they’re like, “Yeah, I’m cool with that,” to me, that’s consent.

However, participants often only gained consent once and then assumed ongoing consent from their partner’s bodily responses to different sexual acts, constituting a form of interpersonal communication. Elijah stated:

I always mention at some point, “if I’m doing something that you don’t like, let me know,” but I don’t ask, “can I do this? Now can I do this?” Once [sex] initially starts, it’s going with the vibe and what’s feeling good.

Noah also said:

I am always, like, making sure everything’s okay [with female partners] through, you know, verbal communication, asking like, “Is everything okay? How does this feel? Can we go further?” With guys, I feel more like in the attitude of not like the absence of a no means yes, but I would definitely look for cues that they’re not okay with something. I find the physical indications that they’re not okay with it more of a determinant of like they might not consent to what I’m about to do.

Only one participant did not report explicitly obtaining consent from male sexual partners. Instead, Kevin, who was the only participant in his 70s, acknowledged that he often assumes consent from others based on their geographical location and/or lack of rejections to his advances, suggesting location is an important element of sexual cultural scripts and that lack of negative feedback influences interpersonal scripts. Specifically, Kevin shared:

I’m making assumptions that if they’re at a cruise park or if they’re talking to me and I’m making suggestions or I’m looking at their crotch or I’m coming on to them…You know, they get an idea of what’s going on…If I get any kind of a sense that no, they’re not interested, fine. I’m not going to pursue. I’m not going to persist.

Further, although most men reported having explicit conversations about sexual desires and consent, there was a lack of consistency related to whether participants asked sexual partners about their sexual histories. For instance, when recalling their most recent sexual encounter with another man, Caleb noted, “in fairness, I guess neither of us asked each other if you are sleeping with other people.” Meanwhile, Gabe mentioned they directly ask potential sexual partners about their sexual health. Gabe said:

I bring it up. I say, “…I was tested. My results were negative. When was the last time you were [tested]?” And most people are very easy to talk to. You just have to ask the questions, and they’ll usually answer them.

### Theme three: Poor communication with women

The third theme focused on sexual communication with women. Participants reported poorer sexual communication with women compared to with men, suggesting that a cultural force related to gender is in play. In particular, participants shared that they talked differently with men versus women, often utilizing several communication strategies with men. Meanwhile, they relied heavily on implicit and nonverbal communication strategies with women, influencing interpersonal sexual scripts. Indeed, although participants often had explicit conversations about sex with other men, they were usually less inclined to have clear and open conversations about sex with women. In fact, only Caleb and Gabe said obtaining consent was similar for men and women. Gabe stated, “[it’s the] same conversation if you ask me. Although, in my opinion, the consent conversation only really needs to happen the one time.”

The remaining participants all discussed how sexual communication with women was different than sexual communication with men. More specifically, participants explained that sexual relationships with men have focused solely on sex while sexual relationships with women stem from romance. According to the participants, this allowed them to feel more comfortable explicitly discussing sexual interests with men. Quinn shared:

Men are more—and I’m sure it’s not accurate now that I’m saying it out loud—but men are more looking for sex, so I’m more upfront. This is my line: “What are you looking for?” Whereas when women are talking to guys—and, again, now that I say this out loud, this doesn’t sound right—but when women are talking to guys, they’re looking more for the relationship.

Similarly, Alex mentioned that limited sexual communication with women may relate to their Chinese cultural upbringing. Alex explained:

I know what I want, what makes me happy. For example, I can ask the men to give me, for example, a blow job or a massage, which makes me happy. But with girls, I don’t ask them to do that. With my wife, or with my ex-girlfriends, maybe this is about cultural things. Yeah, usually we should just do the traditional intercourse. But with men, it’s okay to ask.

Alex later continued, rationalizing the limited sexual communication with his wife because sex with his wife was about getting pregnant and not about seeking pleasure. This represents both intrapersonal and cultural understandings of the purpose of sex between men and women. Alex stated:

Before our baby was born, the purpose for sex is to have a baby. It is not about the physical happiness, so we just say this is about appointment. For example, on Saturday or Friday night, once we feel relaxed, we say, “let’s go to appointment.” So, no need to do some initial communication because we know our purpose to have sex.

Furthermore, for one participant, a lack of clear communication about sex with women seemingly translated to a lack of communication about consent. Kevin recalled one sexual encounter with a woman that involved not having a conversation about consent and not listening to her. Kevin said:

There was a time when I seduced a girl in university, and she kept saying no and I didn’t listen. And I think in hindsight, looking back, I probably would admit that at the end of the day, I raped her…I can’t kid myself that that isn’t what it was. You know, I mean, she resisted enough and said no. I just wasn’t going to accept that.

Kevin was the same participant that acknowledged lacking explicit conversations about consent with men and was the only participant above the age of 70. Thus, the lack of conversations about consent may be specific to Kevin or his age group and may not be reflective of other H-MSM in the present study. Nevertheless, most participants still had clear and explicit conversations about sex with men but not with women.

## Discussion

The present study investigated the sexual communication strategies of eleven H-MSM. This sample of H-MSM shared stories of how they have identified sex partners and how they have communicated about sex with men versus women, revealing stark differences in gender-based sexual communication strategies. These novel findings have important implications for sexual health and well-being as well as interpersonal violence prevention.

Findings from the present study align with previous research that has reported H-MSM meet other men for sex in various sexualized venues (e.g., bathhouses, saunas, cruising parks) and via the internet (Scheadler et al., 2024). Indeed, MSM commonly leverage virtual platforms to directly communicate their sexual desires, preferences, and boundaries with potential sexual partners (Suenzo, 2024). Perhaps meeting via dating apps, such as Grindr, minimizes discomfort with discussing sexual interests, and transforms sexual scripts from implicit and nonverbal and explicit and verbal. Specifically, Grindr and other dating apps allow H-MSM to maintain discretion by adopting fake names and sharing limited information on their profiles. By maintaining discretion, H-MSM may be able to avoid cultural, interpersonal, and intrapersonal pressures to comply with traditional heterosexuality and masculinity, making sexual diversity and adventurousness feasible and facilitating clear sexual communication.

In addition, Grindr and other dating apps and sexualized MSM environments normalize sexual exploration and interactions (Dietzel, 2022; Suenzo, 2024), potentially creating alternative sexual scripts, which is important in promoting sexual agency and communication (Rittenhour & Sauder, 2024). Thus, H-MSM in the present study may have felt more comfortable with explicit sexual communication about their interests when the threat of being stigmatized is minimized and the acceptance of sexual communication is maximized.

Similarly, explicit sexual communication may not feel stigmatizing when sexual partners are perceived as sexual beings opposed to romantic partners. According to sexual script theory, cultural, interpersonal, and intrapersonal pressures influence how individuals, including H-MSM, engage in and talk about sex (Simon & Gagnon, 1984). There is a possibility that a focus on discreet and casual sexual relationships relieve some of these pressures, permitting explicit sexual communication to be acceptable. However, this contradicts prior research that has found that explicit verbal communication is most common in sexual relationships with romantic partners (Marcantonio et al., 2022). Perhaps for H-MSM, the nature of a casual sexual relationship allows sexual scripts to be transformed and norms to be shifted towards clear sexual communication. Put differently, placing a primary focus on sex may make explicit sexual communication feel more natural, comfortable, and logical whereas a focus on emotional and romantic connections may make H-MSM feel pressured to comply with society’s expectations for relationships.

Moreover, extant literature has argued that heterosexual men rely on nonverbal cues from women and adhere to societal expectations around masculinity (Beres et al., 2004; Masters et al., 2013; Waling, 2024; Webber et al., 2024) even when they are critical of those norms and expectations (Murray, 2018). Gay and bisexual men also rely heavily on nonverbal cues for sexual communication and consent (Beres et al., 2004; Webber et al., 2024). Based on results from the present study, H-MSM may similarly emphasize implicit and nonverbal sexual communication and uphold hypermasculine ideals—at least with women. Indeed, the participants described a reliance on implicit and nonverbal sexual communication with women but explicit and verbal sexual communication with men.

Research on sexual script theory, which suggests sexual scripts are gendered (Byers, 1996; Mosher & Tomkins, 1988), may be helpful for explaining these gendered differences. Men are often expected to be dominant over women (Sanchez et al., 2012) and are expected to hold greater sexual agency, determining when sex occurs (Wiederman, 2005). Notably, holding greater sexual agency does not mean men are more likely to utilize explicit and verbal sexual communication strategies. Instead, this means that men are expected to initiate sex and determine how sex proceeds, which could be done via implicit and nonverbal cues. Meanwhile, women’s sexual agency and health is often considered taboo and goes undiscussed (Tohit & Hague, 2024). Women also are expected to be more emotionally driven and care less about sex than men, who are expected to freely express themselves sexually (Levant et al., 2012; Schnarrs et al., 2012). Thus, in order to uphold these standards and expectations, H-MSM may utilize and rely on implicit sexual cues and consent when interacting with women as if discussing sex with women would diminish a man’s sexual agency and as if women have little interest in sexual pleasure. This also may potentially lead H-MSM to perceive other men as beholders of greater sexual agency and interests. One participant in the present study referred to sex with their wife as a means to build a family while sex with men is about seeking pleasure. Therefore, as expected, sexual scripts appear to be limiting sexual communication between some H-MSM and women while permitting more open sexual communication with men.

Finally, the belief that consent is only needed in one instance must be addressed. Consent is an ongoing process and consistent explicit agreement to engaging in various sexual acts is useful for ensuring sexual contact does not become nonconsensual (Glace et al., 2021). The lack of interest in securing ongoing consent with sexual partners may suggest that H-MSM may be more prone to engaging in nonconsensual sex, especially with women whom they communicate with less than with men. Indeed, acceptance and use of implicit nonverbal communication strategies as a form of consent has been linked to self-reported sexual assault perpetration (Walsh et al., 2021). Likewise, some participants also reported believing consent is implied in certain contexts, such as when visiting cruising parks. However, although sexual consent should be contextualized, visiting a sexualized geographical or virtual setting is not synonymous with providing consent to any or all forms of sexual contact. Research has begun to explore multiple motivations for Grindr usage such as platonic socializing with other MSM (Aitken & Taylor, 2024). As such, individuals should still seek explicit verbal consent.

### Implications

This study has several implications for future practice. More work is needed to destigmatize sex and encourage conversations about sexual health and interests. Sex is often considered a taboo subject, resulting in a lack of explicit sexual communication (Tohit & Hague, 2024). Yet, positive communication patterns can enhance sexual desire and satisfaction (Leistner et al., 2024; Mark & Jozkowski, 2013), reduce risky sexual behaviors (Ayer et al., 2021), and strengthens HIV treatment adherence (Jin et al., 2021). Future research and interventions should focus on empowering H-MSM to initiate explicit conversations about sexual health and desires with all their partners, regardless of sex, gender, or setting. Explicit conversations about sexual health and desires may strengthen sexual satisfaction and facilitate safe exploration of sexual desires. Therapeutically, challenging H-MSM’s identity-behaviour discordance may lead to rupture and disengagement (Scheadler et al., 2024) but challenging gendered notions of sexual scripts may facilitate consensual sexual communication.

Relatedly, dating and hookup apps for MSM seem to facilitate and normalize sexual communication between MSM of various identities (Dietzel, 2022; Suenzo, 2024). Researchers and practitioners may consider collaborating with these dating and hookup apps to include educational features that teach users about consent and healthy forms of sexual communication. For example, pop-ups and banners can be used to provide brief facts about consent and tips on how to communicate about sexual desires.

Additionally, sexual health education should be more inclusive of alternative sexual scripts, which can be leveraged to encourage sexual agency and communication (Rittenhour & Sauder, 2024). For example, alternative sex scripts can combat traditional sex scripts that emphasize heterosexual men’s agency. Encouraging heterosexual men to actively consider and listen to the sexual desires of women may facilitate conversations related to consent and sexual desires and boost sexual satisfaction.

### Limitations

There are several limitations worth noting. As with many studies that utilize IPA, the sample was relatively small and homogenous. Also, data related to city and province were not collected. Data related to class information, student status, and disability were also not collected. Likewise, Kevin was the only participant who was over the age of 70 and was the only participant who self-reported sexually assaulting a woman. Kevin’s experience may relate to the beliefs about sex and consent among others in his generation and may not be generalizable to other H-MSM. There are possibilities that H-MSM from different cultural and age backgrounds have different experiences related to their sexual communication patterns. Additionally, participants were recruited from social media advertisements, posts to online forums, and flyers in HIV clinics. There are possibilities that some H-MSM were not reached through these methods. Future research should consider utilizing multiple recruitment strategies to include a diverse sample of H-MSM to help uncover additional cultural differences and to expand upon the findings in the present study. The study was only available in English and required access to technology. Finally, the present study relied upon self-reported sexual communication patterns. There are possibilities that their sexual partners had different perceptions of their sexual communication. Future research could consider studying interpersonal sexual communication between H-MSM and their sexual partners; for example, reviewing text communications between H-MSM and other men on dating and hookup apps.

## Conclusion

Findings revealed that H-MSM use different sexual communication strategies with men than they do with women, suggesting that their sexual communication strategies are gendered. Specifically, H-MSM rely on explicit verbal and written communication strategies to convey their sexual desires and obtain consent with men. However, they rely more heavily on implicit sexual cues when engaging in sex with women. These results have implications for future research and practice related to enhancing sexual satisfaction and ensuring all sexual encounters are consensual.
